# Harnessing selenium nanoparticles (SeNPs) for enhancing growth and germination, and mitigating oxidative stress in *Pisum sativum* L.

**DOI:** 10.1038/s41598-023-47616-5

**Published:** 2023-11-21

**Authors:** Job T. Tendenedzai, Evans M. N. Chirwa, Hendrik G. Brink

**Affiliations:** Department of Chemical Engineering, Faculty of Engineering, Built Environment and Information Technology, University of Pretoria, Pretoria, 0002 South Africa

**Keywords:** Biotechnology, Environmental sciences, Engineering, Nanoscience and technology

## Abstract

Selenium, an essential micronutrient for plants and animals, can cause selenium toxicity as an oxyanion or at elevated doses. However, the toxic selenite (SeO_3_^2−^) oxyanion, can be converted into less harmful elemental nano-selenium (Se^0^), with various practical applications. This research aimed to investigate two methods for reducing SeO_3_^2−^: abiotic reduction using cell-free extract from *Enterococcus* spp. (abiotic-SeNPs) and chemical reduction involving L-ascorbic acid (chemical-SeNPs). Analysis with XPS confirmed the presence of Se^0^, while FTIR analysis identified surface functional groups on all SeNPs. The study evaluated the effects of SeO_3_^2−^, abiotic-SeNPs, and chemical-SeNPs at different concentrations on the growth and germination of *Pisum sativum* L. seeds. SeO_3_^2−^ demonstrated detrimental effects on germination at concentrations of 1 ppm (germination index (GI) = 0.3). Conversely, both abiotic- and chemical-SeNPs had positive impacts on germination, with GI > 120 at 10 ppm. Through the DPPH assay, it was discovered that SeNPs exhibited superior antioxidant capabilities at 80 ppm, achieving over 70% inhibition, compared to SeO_3_^2−^ (less than 20% inhibition), therefore evidencing significant antioxidant properties. This demonstrates that SeNPs have the potential to be utilized as an agricultural fertilizer additive, benefiting seedling germination and development, while also protecting against oxidative stress.

## Introduction

Selenium (Se) is an essential microelement that can be both essential and toxic to living organisms. This is generally determined by its dosage and type of Se species present in the source^1^. The World Health Organisation (WHO) recommends a safe dosage of at most 40 μg Se/L in drinking water^2^. When it comes to the Se species, selenite (SeO_3_^2–^) is its most toxic oxyanion^3^ in addition to being highly reactive^4^. On the contrary, elemental Se (Se^0^) is naturally insoluble, and it can form elemental Se nanoparticles (SeNPs) which have very good bioavailability^5^ and display low toxicological potential^5,6^.

Se is naturally occurring hence its interaction with plants through air, aquatic systems, and the soil is inevitable. Although it is essential for plant development, constant exposure to high Se concentrations leads to its accumulation in plants^7^. This could have toxic effects, affecting the plant growth as high Se concentrations alter the structure and behaviour of proteins^8,9^. Moreover, a variety of biochemical processes and physiological processes can be affected through altering the uptake, aggregation, and transit of mineral nutrients^10^.

In contrast, Se has many positive effects on plants in moderation. Examples include growth promotion and modulation of oxidative stress (antioxidant)^7^. Antioxidants work by blocking the oxidation processes through the neutralisation of free radicals. This neutralisation can be done in two ways; either by chain-breaking or preventative^11^. Se is a component of antioxidant enzymes like thioredoxinreductase^12,13^. Biofunctionalized SeNPs on the other hand, have been found to possess more antioxidant activity than the Se salt, sodium selenite^14^. SeNPs can reduce the accumulation of free radicals or reactive oxygen species (ROS) and prevent an oxidative stress. At high concentrations however, they can be toxic and can contribute to pro-oxidative reactions^7,13^.

In recent years, the interest in metallic or metal nanoparticles has increased. Researchers and scientists are particularly interested in this field because nanomaterials synthesised from noble metals like gold, platinum and silver^15^, as well as from metalloids such as Se and tellurium^16^, have various beneficial properties. They can be useful for agriculture^17^, catalysis^18^, disease diagnosis and treatment^19^, sensor technology, and mammographic instrument detectors^20,21^. In particular, SeNPs have recently attracted more attention in many scientific fields as Se has been widely used in food supplements and nanomedicine^1^.

In agriculture, Se in the form of sodium selenite can be used as an additive in fertilizers^21^ because it may act as a quasi-essential micronutrient through altering different physiological and biochemical traits^22^. However, when it comes to standard fertilizer, the application of Se as nano-Se is more efficient than Se in its inorganic form when looking at biological processes and yield in the soil and plants^23^. Therefore, with the emergence of nanotechnology, the use of SeNPs as an adjunct to standard Se fertilizers to improve crops has emerged as a feasible option to conventional Se fertilizers^7^.

Although Se is ubiquitous and has several natural sources, anthropogenic sources of late have been a major source of Se pollution in water sources. The mining industry, in particular, is one of the major contributors of Se release into the environment^24^. Examples of such operations include the mining of precious metals like silver and gold as well as coal. Waste rock disposal and tailings produced during the mining and processing of metallic ores are a source of the release of Se into the environment^25^. In the case of coal, burning it produces ash enriched with Se which subsequently leaches due to rainfall thereby contaminating nearby aquatic systems^26^.

Therefore, Se from such sources must be removed from aquatic systems and could therefore be recovered for potential reuse. Different physical and chemical methods such as electrochemical changes, chemical reduction, and photochemical reduction are commonly employed for the preparation and stabilization of metallic nanoparticles^27^. In this study, two methods were used to reduce selenite to elemental Se. Firstly, abiotically (abiotic-SeNPs) using secretions from a biological catalyst (*Enterococcus* species) as described by Tendenedzai et al.^6^ and secondly, by chemical means (chemical-SeNPs), using a reducing agent (L-ascorbic acid) as described by Shahabadi et al.^28^. Once the nano-Se had been recovered, the effect of different concentrations of SeO_3_^2−^, abiotic-SeNPs, and chemical-SeNPs on the germination of *P. sativum* (pea seeds) was tested. Moreover, the nanoparticles’ antioxidant capacity was investigated using 2,2-diphenyl-2-picrylhydrazyl hydrate (DDPH).

## Materials and methods

### Chemicals, culture media, and solutions

All chemicals used were from Sigma-Aldrich (St. Louis, MO, USA) unless otherwise specified.

### Culturing, growing, and concentrating the selenite-reducing bacteria

The bacterial cultures used in the present work were *Enterococcus* spp. isolated from Se-laden medium found in a laboratory at the University of Pretoria South Campus, South Africa. In a previous study conducted in 2021, the characterization of *Enterococcus* spp. was carried out using a range of analytical techniques, including TEM (Transmission Electron Microscopy), SEM (Scanning Electron Microscopy), and 16s rRNA sequencing^29^. For activation, the bacteria were aerobically cultivated in fresh Tryptone Soya Broth (TSB) (Oxoid Ltd., Basingstoke, UK) on a rotary shaker (Labotech, Midrand, South Africa) (28 °C, 24 h, 120 rpm). Thereafter, they were concentrated and harvested by centrifugation (6000 rpm, 25 °C, 5 min) before being utilised in the reduction experiments.

### Abiotic-SeNPs and chemical-SeNPs synthesis

Sodium selenite (Na_2_SeO_3_) was prepared as a 100 mM stock solution (stabilised with 300 mM NaOH) and SeO_3_^2−^ was added as Na_2_SeO_3_ with an initial selenite concentration of 2 mM. Abiotic-SeNPs were prepared using a method from an earlier study^6^ in which the aerobic batch reduction was carried out in two stages: firstly, in the presence of bacterial biomass (biotic stage) for 1 h and secondly, in the absence of biomass by use of the cell-free extract (abiotic stage). The mineral salt medium (MSM) was similar to the one used elsewhere^29^. The starting pH was between pH 8.5–9.5; the temperature was maintained at 35 ± 2 °C; the rotary speed was 120 rpm. The total experiment run time was 96 h.

**Figure 1:**
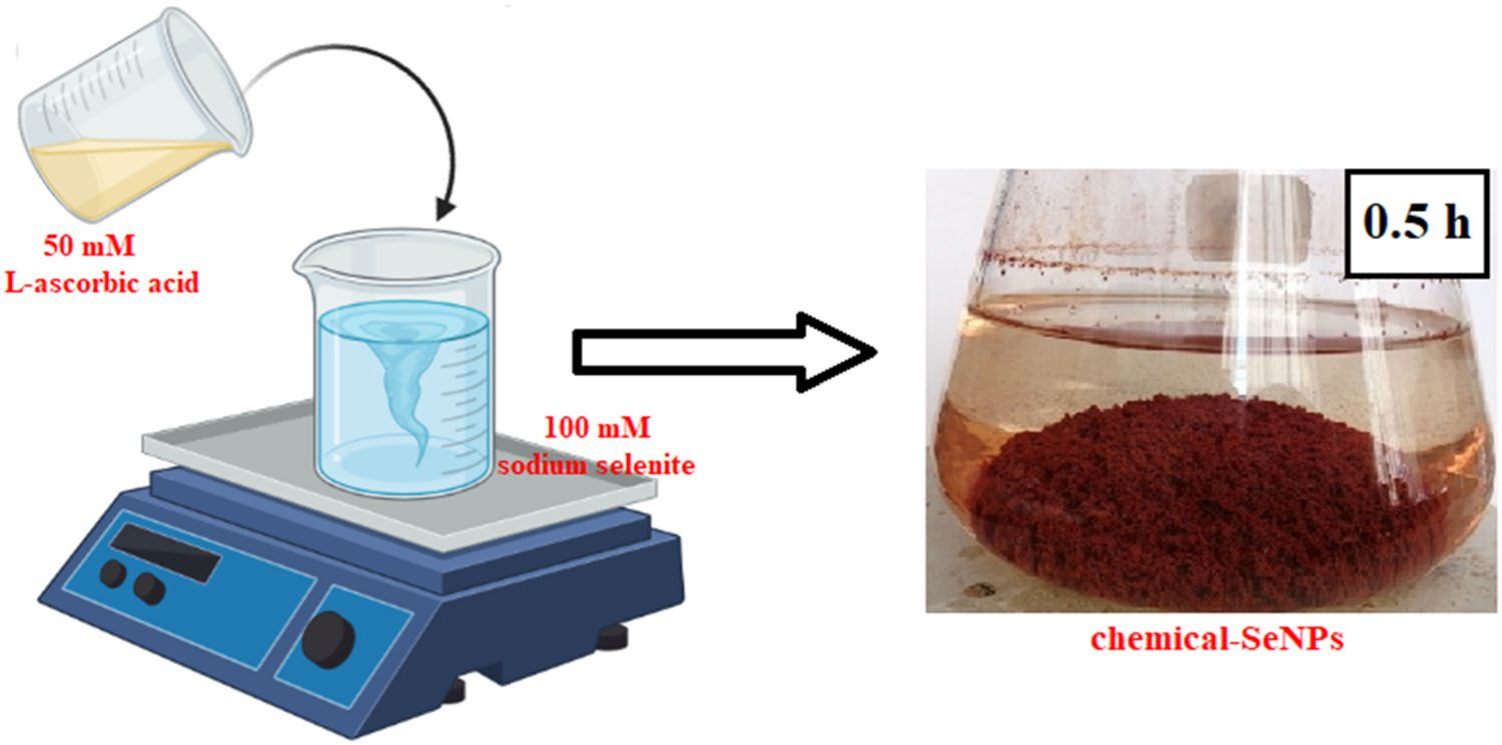
Chemical reduction of SeO_3_^2−^ by ascorbic acid (partially created with https://www.biorender.com/).

The chemical-SeNPs were prepared by reacting SeO_3_^2−^ with L-ascorbic acid (C_6_H_8_O_6_) in a one-step reaction. Figure 1 below depicts the one step reaction in the formation of the chemical-SeNPs using L-ascorbic acid (C_6_H_8_O_6_) as a reductant. Specifically, 50 mM C_6_H_8_O_6_ was added dropwise to an aqueous solution containing 100 mM Na_2_SeO_3_. The volume ratio for sodium selenite to ascorbic acid was approximately 1:2. The reaction was fairly rapid and upon addition of the reductant into the sodium selenite, the system was gently stirred at room temperature and the solution turned into light yellow and eventually into dark red after 30 min.

After 30 min the nanoparticles were collected by centrifugation (11,000 rpm, 25 °C, 15 min.). The residue was washed twice with ethanol followed by deionized water to remove excess ascorbic acid and other by-products. The collected particles were air dried for 24 h and stored in air-tight bags for subsequent experiments.

For both synthesis methods, the selenite concentration was measured in the supernatant using the 940 Professional IC Vario ion chromatograph (Metrohm, Herisau, Switzerland) with separation column Metrosep C 6-250/4.0 (Metrohm, Herisau, Switzerland) and C 6- eluent- 8 mM oxalic acid (Metrohm, Herisau, Switzerland). Selenite which had been reduced to elemental Se nanoparticles was concentrated in the pellet from centrifugation. For it to be quantified, it was first resuspended with 0.1% saline and washed before acid digestion (70% HNO_3_, 32% HCl, 60 min, 100 °C) in a thermo-reactor (Spectroquant^®^, Sigma-Aldrich, St. Louis, MO, USA).

Total Se in the digested sample was determined using Varian AA–1275 Series Flame AAS (Varian, Palo Alto, CA, USA) at 196.03 nm wavelength equipped with a 290 mA Se lamp. All experiments were done in triplicate unless otherwise stated. Some pellet samples were collected for further characterisation. These were purified by sequential centrifugation (10 000 rpm, 10 min) in 0.1% saline solution carbon-free, distilled, deionized water.

In summary, while using bacteria, there were two stages in which SeNPs were synthesised, i.e., biotic stage (1 h) and abiotic stage (95 h). The biotic stage was faster because of the presence of the biomass which; (i) rapidly reduced the high selenite concentration as a detoxication mechanism and, (ii) also secreted metabolites which aided is faster selenite reduction and SeNPs formation. For abiotic synthesis, SeNPs formation was predominantly reliant on selenite-reducing metabolites present in the medium, once the biomass had been removed, which is why it was not as rapid^30,31^. For chemical synthesis, a one-step synthesis method was used and it took 30 min to form the SeNPs.

### XPS and FTIR analysis

X-ray photoelectron spectroscopy (XPS) was used to characterize the abiotic-SeNPs with a Thermo ESCAlab 250 Xi (Madison, USA). Samples were rinsed with distilled water after reduction experiments and dried at room temperature before analysis.

The PerkinElmer Spectrum 2000GX FTIR spectrometer (Perkin Elmer, Waltham, USA) using an attenuated total reflection (ATR) attachment was used for FTIR analysis on the abiotic-SeNPs and the FTIR spectrum was obtained in the wavenumber range of 4000–650 cm^−1^.

### Phytotoxicity/phyto-benefit assay on pea seeds (*P. sativum*)

Pea seeds (*P. sativum*) supplied by STARKE AYRES® (Hartbeesfontein, North West, South Africa) were utilized in the assay. Each sterilised Petri dish (1 × 10 cm) contained 10 seeds and approximately 4 g of rockwool growing medium obtained from Hydroponic.co.za (Claremont, Western Cape, South Africa). The seeds were treated with 30 ml test solutions at various concentrations (0 ppm, 0.1 ppm, 1 ppm, 10 ppm, and 100 ppm) of selenite, abiotic-SeNPs, or chemical-SeNPs. After 7 days of incubation in light at 25 °C, the percentage of seed germination, number of roots, root length, and shoot length were determined. The experiments were conducted in triplicate, resulting in a sample size of 30 seeds per condition. The relative seed germination, relative root length, and germination index were calculated using the provided equations. This was done aiming to assess the potential of nano-selenium as a fertilizer additive to enhance seedling while assessing the impacts of selenite-induced phytotoxicity.

To assess the impact of selenite, abiotic-SeNPs, or chemical-SeNPs on the growth and biomass accumulation of germinated peas, wet and dry weight experiments were conducted. Germinated seeds were incubated in light at 25 °C with varying concentrations of the test solutions for 7 days. After incubation, the seeds were carefully harvested, and each seedling was immediately weighed without removing any moisture, representing the wet weight. The seeds from each test solution group were then dried in an oven at 80 °C for 24 h. Once dried, the seeds were cooled in a desiccator at room temperature and weighed to record the dry weight data.

1 $$\mathrm{Relative\,seed\,germination }(\mathrm{\%})=\frac{\mathrm{number\,of\,seeds\,germinated\,in\,test\,solutions}}{\mathrm{number\,of\,seeds\,germinated\,in\,control}}\times 100$$

2 $$\mathrm{Relative\,root\,length }(\mathrm{\%})=\frac{\mathrm{mean\,root\,length\,in\,test\,solutions}}{\mathrm{mean\,root\,length\,in\,control}}\times 100$$

3 $$\mathrm{Germination\,index }(\mathrm{\%})=\frac{\mathrm{ \% seed\,germination }\times \mathrm{\% root\,length} }{100\mathrm{ \%}}$$

### Antioxidant activity of SeO_3_^2−^ and SeNPs selenite using the DPPH assay

The antioxidant potency of SeO_3_^2−^, abiotic-SeNPs, and chemical-SeNPs was measured using DPPH (2,2-diphenyl-2-picrylhydrazyl hydrate) analysis. This is a DPPH radical scavenging assay similar to the one published elsewhere^32^. 5 ppm, 10 ppm, 20 ppm, 40 ppm, and 80ppm of either SeO_3_^2−^, abiotic-SeNPs, and chemical-SeNPs were used. The solutions (prepared in DMSO) to be investigated were mixed with 2 mL of a 0.2 mM solution of DPPH in a methanol solvent and mixed well. Thereafter, the mixture was incubated for 30 min in the dark. The absorption of the samples was detected at 517 nm using a UV–vis. L-ascorbic acid (C_6_H_8_O_6_) was used as the standard. The equation for measuring antioxidant activity, similar to the one described by Al Jahdaly et al.^33^ is indicated below;

4 $$\mathrm{Inhibition\,percentage\, of\, DPPH}=\frac{\mathrm{the\,absorbance\,of\,control }-\mathrm{absorbance\,of\,sample}}{\mathrm{absorbance\,of\,control}}\times 100$$

### Ethical statement

The seeds of *Pisum sativum* L. used in this study were purchased from STARKE AYRES^®^ (Hartbeesfontein, North West, South Africa). No endangered species were used in this study. No special permissions or permits were required to source or purchase the seeds. The experimental procedures were performed and complied with relevant institutional, national, and international guidelines and legislation.

## Results and discussion

### Abiotic SeO_3_^2−^ reduction and SeNPs formation

**Figure 2:**
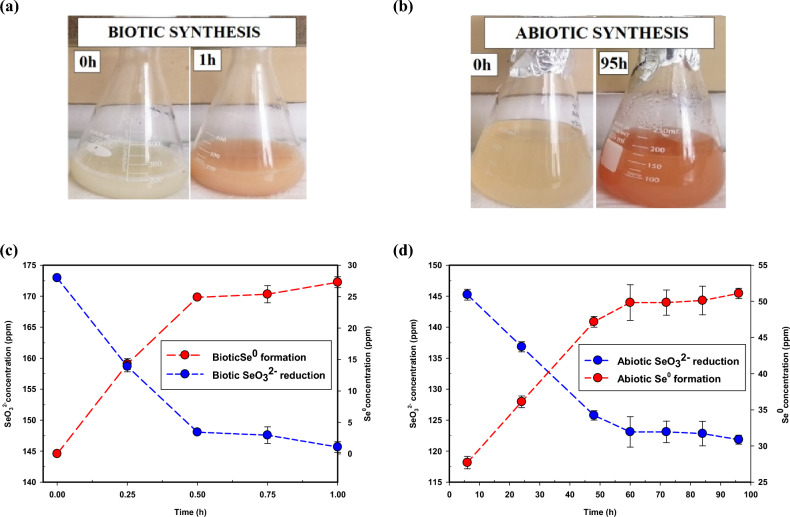
Colour change in (**a**) Biotic and (**b**) Abiotic reduction of SeO_3_^2−^, Profiles for (**c**) Biotic reduction and formation and (**d**) Abiotic reduction and formation.

The abiotic-SeNPs were synthesized through aerobic reduction as described earlier in two stages, i.e., with biomass for 1 h and abiotically for the remaining 95 h. The initial SeO_3_^2−^ concentration at time 0 h was 172 ppm (≈ 1mM). Figure 2(a), (b) show the change in colour between the two stages. Reddish abiotic-SeNPs were observed after 95 h as shown in Fig. 2(b) similar to what was observed by Tendenedzai et al.^6^. The SeO_3_^2−^ reduction and SeNPs formation profiles are shown in Fig. 2(c), (d) respectively and the stage with biomass was faster than the abiotic stage.

However, the continual formation of SeNPs in the abiotic stage indicated the presence of selenite reducing metabolites which form nano-Se extracellularly as suggested by Saima Javed et al.^34^. The average concentration of SeNPs recovered for the entire process was 51.08 ± 0.71 ppm and of which, 23.81 ± 1.27 ppm was entirely from the abiotic stage. This accounts for almost half of the total. The advantage of being able to recover SeNPs from an abiotic system is that it circumvents the problem of having to separate the nanoparticles from bacterial biomass which can be costly^6^.

### Abiotic-SeNPs identification and characterisation

**Figure 3:**
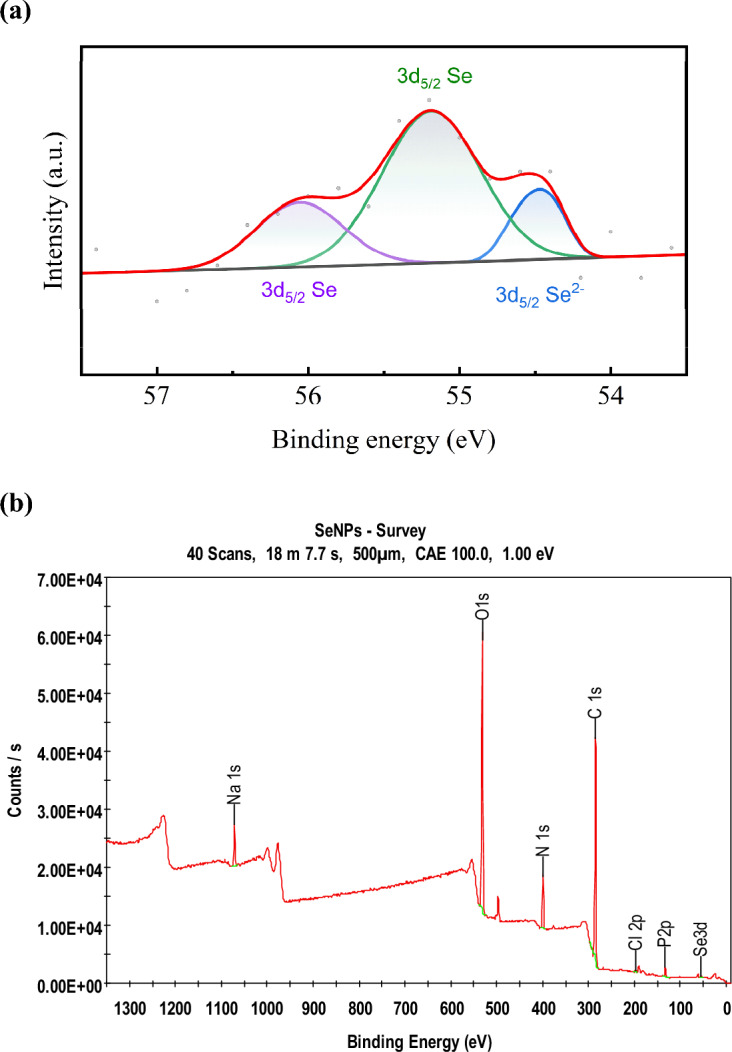
XPS spectra of Abiotic-SeNPs: (**a**) wide-scan spectra, (**b**) high resolution Se 3d_5/2_ spectra.

The XPS analysis revealed that the abiotic-SeNPs sample consisted of a mixture of polarized Se^0^ and Se^2−^. This conclusion was supported by the composition of the Se 3d peak observed in Fig. 3. Previous research by Castle^35^ stated that Se^0^ is expected at a binding energy of 55.1 eV for the Se3d_5/2_ peak, while Se^2−^ is expected at lower binding energies. Our findings align with these expectations. Additionally, it is worth noting that polarized Se^0^ can sometimes exhibit slightly higher binding energies, as reported by Rupp and Weser^36^. In our analysis, the binding energies measured at 55.2 eV and 56 eV in the sample confirmed the presence of Se^0^. However, it is important to acknowledge that a portion of the detected Se signal might be attributed to the outer organic layer derived from the cell-free extract, as reported by Ruiz-Fresneda et al.^37^.

This organic layer is believed to cover the SeNPs, which is supported by the presence of C, N, P, and O in the survey spectra denoted by the C1s, N1s, P2p and O1s peaks respectively (Fig. 3b) with high carbon (C1s) and oxygen (O1s) counts^38^. These peaks provide information about the chemical environment and bonding of these elements within the material being analysed. The C1s peak typically arises from carbon-containing functional groups or organic compounds present in the sample (C–C, C–H, or C–O bonds)^38^. N1s peak originates from the nitrogen atoms within the sample and indicate the presence of nitrogen-containing species, which could include amines, amides, or other nitrogen-based functional groups. The P2p peak corresponds to the binding energy of phosphorus core electrons. Its presence indicates the presence of phosphorus-containing species or compounds. The O1s peak represents the binding energy of oxygen core electrons and it reflects the presence of oxygen-containing functional groups or compounds within the material (C–O, C=O, or O–H bonds)^38^.

**Figure 4:**
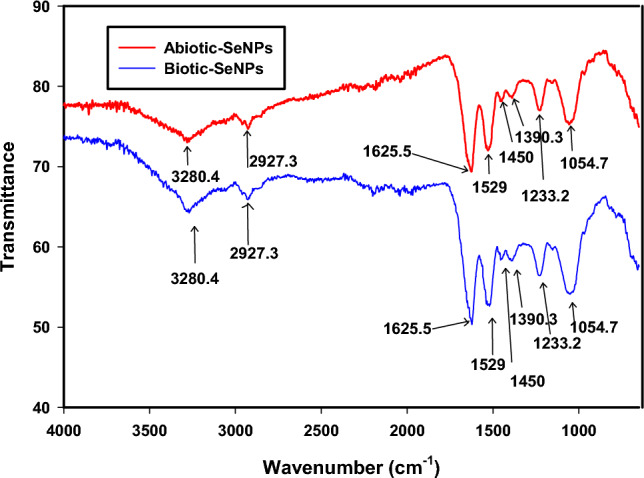
FTIR spectrum of Biotic and Abiotic-SeNPs.

FTIR analyses was consonant with the results for the XPS and confirmed the presence of organic materials on the surface of both the biotic-SeNPs and abiotic-SeNPs as shown in Fig. 4 above. The presence of functional groups was confirmed, indicating the likely presence of macromolecules such as lipids, sugars, carbohydrates, and nucleic acids^39^. The peaks were obtained between 4000 and 650 cm^−1^. The identities of the peaks are summarised in Table 1. The table was adapted from a previous study by Tendenedzai et al.^6^. The results confirmed that the biotic and abiotic nanoparticles displayed the same functional groups.

**Table 1:** Wavenumbers of the main bands in the FTIR spectrum of biotic and abiotic SeNPs.

Wavenumber (cm^−1^)	Functional groups	References
3280.4	N–H_2_, aminoacidic group	^40^
2927.3	C–H, C–H_2_ stretch, Alkanes, aliphatic groups, fatty acid aliphatic chains	^41^
1625.5	N–H stretch, Secondary amine, amide I	^42^
1529	C–N stretch, amide II band, alkanes	^40^
1450	–CH_2_/–CH_3_ (in proteins, lipids, polyesters, etc.)	^40,43^
1390.3	Carboxyl (–COO–) stretching vibration	^41,44^
1233.2	C–N stretch, amide III band, O–P–O	^40^

The FTIR spectra of the SeNPs exhibited several peaks at specific wavenumbers, each indicating the likely origin of certain functional groups. At the wavenumber of 3280.4 cm^−1^, the peak corresponding to N–H_2_, aminoacidic group suggests the presence of amino acids or compounds containing amino groups, potentially derived from biomolecules present in the cell free extract or microorganisms involved in the bioreduction process^40^. Another peak observed at 2927.3 cm^−1^ is attributed to the stretching vibrations of carbon–hydrogen (C–H) bonds in alkanes, aliphatic groups, and fatty acid aliphatic chains. This peak indicates the presence of hydrocarbon-based compounds or organic molecules with long hydrocarbon chains, which may originate from cellular components such as lipids and proteins^41^. The peak at 1625.5 cm^−1^ corresponds to the stretching vibrations of nitrogen–hydrogen (N–H) bonds in secondary amines and is associated with the amide I band. It indicates the presence of peptide bonds (C=O–NH) in proteins or other compounds containing amide groups. These secondary amines and amide bonds may form from the breakdown of amino acids or other nitrogen-containing compounds during the bioreduction process^42^. At the wavenumber 1450 cm^−1^, the presence of methylene (–CH_2_–) and methyl (–CH_3_) groups suggests the presence of proteins, lipids, polyesters, or other organic compounds containing these functional groups. These functional groups are commonly found in biomolecules and can contribute to the formation of SeNPs during the reduction of selenite^40,43^. The peak observed at 1390 cm^−1^ indicates the presence of organic acids or compounds with carboxyl functional groups. These organic acids, produced by microorganisms or present in the biomass, can play a role in the reduction process and subsequent formation of SeNPs^41,44^. Furthermore, the wavenumber 1233.2 cm^−1^ indicates the presence of amide bonds (N–H–C=O) in proteins or other compounds containing amide groups. These amide bonds can arise from proteins or peptides. The presence of O–P–O suggests the presence of organic phosphates or phosphodiester groups, which could originate from nucleic acids or other phosphorylated biomolecules^40^.

In summary, the identified peaks in the FTIR spectra of Biogenic SeNPs provide insights into the functional groups and biomolecular components involved in the bioreduction process. Amino acids, alkanes, proteins, lipids, organic acids, and phosphorylated biomolecules are likely contributors to the observed peaks, highlighting their roles in the formation of SeNPs.

## Chemical-SeNPs characterisation

The method is comparable to the one from a study by Shahabadi et al.^28^ and yielded similar results^28^. Not all the initial 100 mM selenite was reduced to elemental Se because ascorbic acid has a reducing stoichiometry of 1–2, i.e. 1:2 molar ratio used in the study could not yield total reduction. Instead, substantial aggregation was observed and this is similar to what Liang and co-workers^45^ observed as well. Therefore, approximately 45.34 mM of SeNPs was recovered translating to 7841 ppm.

**Figure 5:**
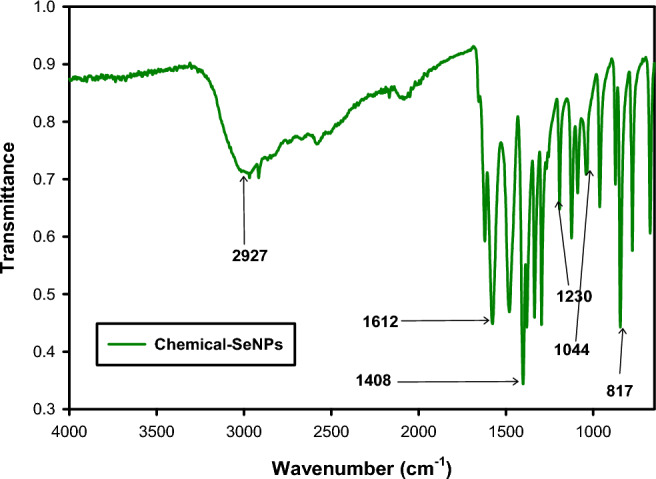
FTIR spectrum of chemical-SeNPs.

The FTIR analysis for the chemical-SeNPs was done between 4000 and 650 cm^−1^ range and the peaks are shown in Fig. 5 and the identities of the peaks are summarised in Table 2.

**Table 2:** Wavenumbers of the main bands in the FTIR spectrum of chemical-SeNPs.

Wavenumber (cm^−1^)	Functional groups	References
2927.3	C–H, C–H_2_ stretch, Alkanes, aliphatic groups, fatty acid aliphatic chains	^41^
1612	C–O stretching vibration	^33^
1408	Secondary NH_2_ group	^33^
1230	Secondary –OH bending	^46^
1044	C–N stretch, amines	^40,47^
817	C–N stretch, amide III band, C–X stretching in alkyl halides	^33,40,47^

At the wavenumber 2927.3 cm^−1^, the peak represents C–H, C–H_2_ stretch vibrations, indicating the presence of alkanes, aliphatic groups, and fatty acid aliphatic chains. These functional groups are commonly found in hydrocarbon-based compounds and can originate from the L-ascorbic acid or other organic molecules involved in the reduction process^41^. The peak at 1612 cm^−1^ corresponds to the C–O stretching vibration, suggesting the presence of carbonyl groups in the SeNPs. This peak may be attributed to the formation of ester or carboxylate groups during the reduction process, possibly arising from the L-ascorbic acid or other organic compounds present^33^. At the wavenumber 1408 cm^−1^, the peak indicates the presence of a secondary NH_2_ group, which suggests the involvement of secondary amines^33^. The peak observed at 1230 cm^−1^ corresponds to the bending vibrations of secondary –OH groups. This peak indicates the presence of hydroxyl groups^46^. At the wavenumber 1044 cm^−1^, the peak corresponds to the C–N stretch vibrations, indicating the presence of amines. These amines may originate from the reduction of L-ascorbic acid or other amine-containing compounds used in the synthesis^40,47^. The peak observed at 817 cm^−1^ corresponds to the C–N stretch vibrations and is associated with the amide III band. This peak suggests the presence of amide bonds in proteins or other compounds containing amide groups. It may originate from L-ascorbic acid or other biomolecules involved in the reduction process. Additionally, the peak at 817 cm^-1^ can also be attributed to the stretching vibrations of C–X bonds in alkyl halides, indicating the presence of alkyl halide impurities in the synthesised SeNPs^33,40,47^.

In summary, the observed peaks in the FTIR spectra of chemical SeNPs synthesized through L-ascorbic acid reduction provide insights into the functional groups and likely sources involved in the synthesis process. Alkanes, carbonyl groups, amines, hydroxyl groups, and amide bonds are among the identified functional groups, indicating the involvement of L-ascorbic acid or other organic compounds in the reduction reaction.

In our study, we found that biotically synthesised SeNPs have simpler surface chemistry and fewer FTIR peaks compared to chemically synthesised SeNPs. This is consistent with findings from Bisht et al.^48^ and Sans-Serramitjana et al.^49^, who noted that chemical synthesis often introduces additional functional groups, leading to more FTIR peaks. Even when using ascorbic acid as a reagent, chemically synthesized SeNPs tend to exhibit more FTIR peaks due to the complex nature of ascorbic acid, which has multiple functional groups^50^. In contrast, biotic synthesis typically results in SeNPs with simpler surface chemistry, often capped with biomolecules containing fewer functional groups, resulting in fewer FTIR peaks.

## Effect of SeO_3_^2−^ and SeNPs on seed germination

**Figure 6:**
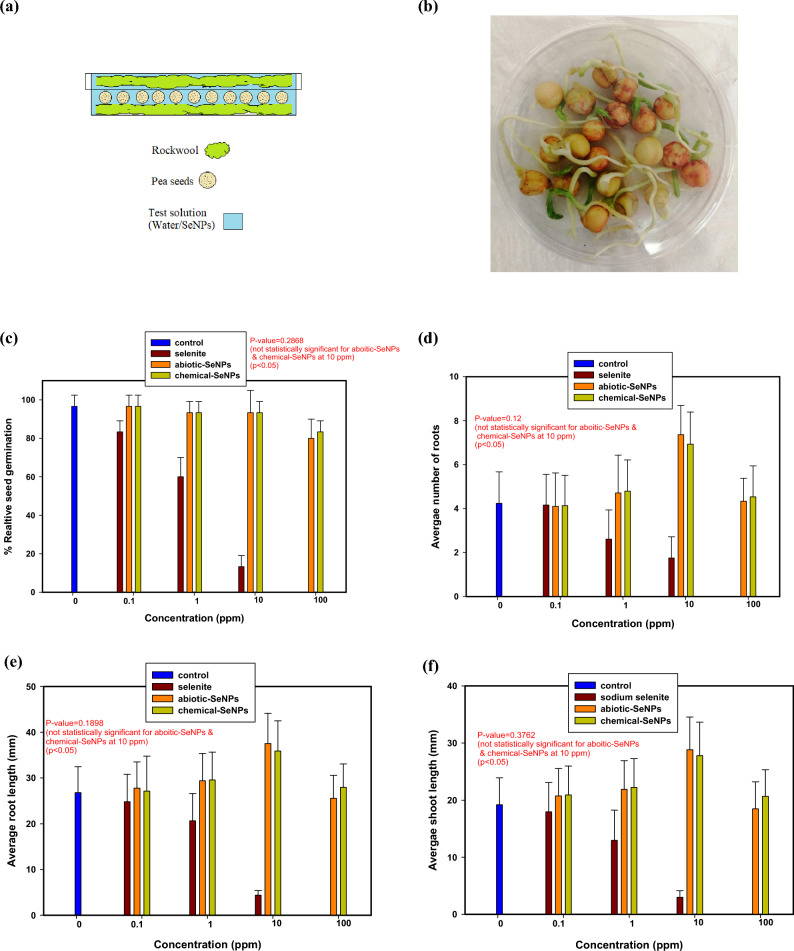
Hydroponic setup; (**a**) cross-section with rockwooland, (**b**) after removal of rockwool; The effect of SeO_3_^2−^, abiotic and chemical SeNPs on (**c**) seed germination, (**d**) number of roots, (**e**) root length and (**f**) shoot length.

Figure 6(a), (b) show the hydroponics setup used for the germination experiments. In this setup, Fig. 6(a) has rockwool growing medium which was used in order to create a conducive environment for growth. The advantage of rockwool is that it can hold large quantities of water or solution as well as air that aid root growth and nutrient uptake in hydroponic systems^51^. The setup ensured that the combination of rockwool and seeds was compeletely soaked in the test solution. Once this had been done, the seeds were allowed to germinate for 7 days. Figure 6(b) illustrates the germinatted *P. sativum* after removal of rockwool.

The effects of the selenite, and Se nanoparticles were tested on pea seeds (*P. sativum*) as previously mentioned, at different concentrations of 0.1 ppm, 1 ppm, 10 ppm and 100 ppm. The result in Fig. 6(c) shows that of all the test solutions, only SeO_3_^2−^ had a significant effect on seed germination with increases in concentration. The control (0 ppm) had a relative seed germination of 96.66 ± 5.77%. However, at just 0.1 ppm SeO_3_^2−^, the relative seed germination notably reduced to 83.33 ± 5.77%. As the SeO_3_^2−^ concentration increased to 1 ppm, 10 ppm and 100 ppm, the relative seed germination plummeted to 60 ± 10%, 13.33 ± 5.77% and 0% respectively. This was an early indication of the negative effects the elevated selenite concentrations were having on the seeds.

Both types of SeNPs had largely positive effects on the seeds and this was indicated by the relative seed germination values. At 0.1 ppm, 1 ppm, 10 ppm and 100 ppm abiotic-SeNPs concentration, the relative seed germination was 96.66 ± 5.77%, 93.33 ± 5.77%, 93.33 ± 11.55% and 80 ± 10% respectively. Likewise, the chemical-SeNPs followed the same trend with the relative seed germination at 96.66 ± 5.77%, 93.33 ± 5.77%, 93.33 ± 5.77% and 83.33 ± 5.77% for the respective concentration range.

The number of roots is shown in Fig. 6(d). The average number of roots per germinated seed in the control was 4.24 ± 1.43. This value drastically reduced with an increase in the SeO_3_^2−^ concentrations. At 0.1 ppm, 1 ppm and 10 ppm, the average number of roots became 4.16 ± 1.4, 2.61 ± 1.33 and 1.75 ± 0.96, respectively. For both the abiotic and chemical nanoparticles, the average numbers of roots were closer to the control for three of the concentrations. At 0.1 ppm, 1 ppm, and 100 ppm abiotic-SeNPs concentration, the average number of roots was 4.1 ± 1.52, 4.71 ± 1.72 and 4.33 ± 1.05, respectively. Similarly, the chemical-SeNPs had the average number of roots at 4.14 ± 1.38, 4.79 ± 1.42 and 4.53 ± 1.41 for the 0.1 ppm, 1 ppm, and 100 ppm, respectively. The highest average number of roots was recorded at 10 ppm and the values were 7.36 ± 1.33 and 6.93 ± 1.46 for the abiotic-SeNPs and chemical-SeNPs, respectively.

The root elongation is shown in Fig. 6(e). The control had an average length of 26.79 ± 5.67 mm. An increase in the SeO_3_^2−^ concentrations hindered root length significantly. The average root length at 0.1 ppm, 1 ppm and 10 ppm SeO_3_^2−^ was 24.78 ± 5.99 mm, 20.62 ± 5.96 mm and 4.38 ± 1.08 mm, respectively. Like the previous parameters, both the abiotic-SeNPs and chemical-SeNPs performed similarly with respect to root elongation. At 0.1 ppm, 1 ppm, 10 ppm and 100 ppm abiotic-SeNPs, the average root length was 27.77 ± 5.71 mm, 29.38 ± 5.96 mm, 37.52 ± 6.61 mm and 25.53 ± 5.04 mm respectively. Similarly, at 0.1 ppm, 1 ppm, 10 ppm and 100 ppm chemical-SeNPs, the average root length was 27.14 ± 7.61 mm, 29.57 ± 6.08 mm, 35.89 ± 6.59 mm and 27.97 ± 5.08 mm, respectively.

The shoot length is shown in Fig. 6(f). The control had an average length of 19.21 ± 4.71 mm. An increase in the SeO_3_^2−^ concentrations also hindered shoot length considerably. The average root length at 0.1 ppm, 1 ppm and 10 ppm SeO_3_^2−^ was 17.95 ± 5.14 mm, 12.97 ± 5.29 mm and 2.98 ± 1.16 mm respectively. Just like root length, both the abiotic-SeNPs and chemical-SeNPs affected the shoot length similarly. At 0.1 ppm, 1 ppm, 10 ppm and 100 ppm abiotic-SeNPs, the average root length was 20.74 ± 4.79 mm, 21.89 ± 5.01 mm, 28.83 ± 5.72 mm and 18.49 ± 4.71 mm respectively. Similarly, at 0.1 ppm, 1 ppm, 10 ppm and 100 ppm chemical-SeNPs, the average root length was 20.91 ± 5.06 mm, 22.21 ± 5.06 mm, 27.79 ± 5.84 mm and 20.766 ± 4.66 mm respectively.

Thereafter, the relative root length was calculated using Eq. 2 and the calculated values were used to determine the germination index (GI). Table 2 shows the germination index (GI) of *P. sativum* after exposure to the different SeO_3_^2−^ and SeNPs concentrations which was calculated using Eq. 3. In essence, the GI summarises the results discussed above. As can be seen in the Table 3, the GI decreased significantly with an increase in SeO_3_^2−^ concentration. For the abiotic-SeNPs and Chemical-SeNPs, the GI was highest at 10 ppm indicating that at this concentration was when the seeds thrived the most.

**Table 3:** Germination index of *P. sativum* after exposure to the different SeO_3_^2−^ and SeNPs concentrations.

Concentration (ppm)	SeO_3_^2−^	Abiotic-SeNPs	Chemical -SeNPs
0 (control)	96.66	96.66	96.66
0.1	66.44	99.49	97.92
1	28.66	98.01	99.22
10	0.3	125	120.73
100	0	62.62	75

The above results put forward a strong argument for the agricultural use of nano-Se as compared to Se oxyanions. A dose–response relationship seemed to exist between the selenite/nanoparticles and seed development, similar to what was observed in a 2015 study on the effects of Se biofortification^52^.

This is evidenced from the results as 10 ppm and 100 ppm SeO_3_^2−^ concentrations completely inhibited germination (GI at these concentrations at 0.3 and zero respectively). High SeNPs concentrations (two orders of magnitude greater), lowered the GI to a similar extent as 1 ppm SeO_3_^2−^, indicating that the concentration range in this study was significantly less toxic than the SeO_3_^2−^. Investigations by a previous study showed results similar to our study in that the Se oxyanions were more harmful to plants than SeNPs^53^.

Statistical analyses were done to determine the significance of all the test solutions on the germination parameters. The first was the Kruskal–Wallis test, which is a nonparametric statistical test equivalent of the one-way ANOVA that assesses the differences among three or more independently sampled groups on a single, non-normally distributed continuous variable^54^. The test with multiple comparisons was applied to check if there are significant differences in the mean; (i) relative seed germination, (ii) number of roots, (iii) root length, and (iv) shoot length, owing to the three test solutions, i.e., the SeO_3_^2−^, abiotic-SeNPs and chemical-SeNPs, relative to the control. The test statistic is defined as:

5 $$H=\left[\frac{12}{n(n+1)}\sum_{j=1}^{c}\frac{{T}_{j}^{2}}{{n}_{j}}\right]-3(n+1)$$

where $n$ = sum of sample sizes for all samples, $c$ = number of samples, ${T}_{j}$ = sum of ranks in the jth sample, ${n}_{j}$ = size of the jth sample.

$${\mathrm{H}0;\overline{x} }_{1}={\overline{x} }_{2}$$

$$\mathrm{H}1:{\overline{x} }_{1}\ne {\overline{x} }_{2}$$

A Kruskal–Wallis Test was performed to determine if the means of the seed germination parameters were the same for the three test solutions at their varying concentrations. In the analyses, a total of 30 seeds were used for each test solution concentration. As seen in Table 4, the relative seed germination did not differ between the test solutions at 0.1 ppm (H = 1.099, *p* = 0.7774), 1 ppm (H = 5.929, *p* = 0.1151) and 100 ppm (H = 1.3457, *p* = 0.5103). This is the same trend which was observed across all the other germination parameters, i.e., the average number of roots, average root length and the average shoot length, respectively. Notable differences were observed at 1 ppm and 100 ppm SeO_3_^2−^ because of the drastic decrease in the values of the germination parameters. The highest values in the germination parameters were observed at 10 ppm SeNPs. As seen in Table 4, there is also no statistically significant difference between the abiotic-SeNPs and chemical-SeNPs. The H-statistic and *p*-values for the relative seed germination (H = 1.1345, *p* = 0.2868), number of roots (H = 1.021, *p* = 0.12), root length (H = 1.7186, *p* = 0.1898), and shoot length (H = 0.783, *p* = 0.3762) confirm this conclusion. This shows that the abiotic-SeNPs (from bacterial excretions) displayed the same properties as the chemical-SeNPs (from L-ascorbic acid). This confirms phyto-beneficial effects of abiotic-SeNPs and chemical-SeNPs as they performed significantly better than the control (0 ppm) and the SeO_3_^2−^ at this concentration.

**Table 4:** Comparison of statistical significance of differences in seed germination parameters across the varying concentrations.

Test solutions	Relative seed germination (%)	Average number of roots	Average root length (mm)	Average shoot length (mm)
Control (0 ppm)	96.66 ± 5.77^a^	4.24 ± 1.43^a^	26.79 ± 5.67^a^	19.21 ± 4.71^a^
SeO_3_^2−^ (0.1 ppm)	83.33 ± 5.77^a^	4.16 ± 1.4^a^	24.78 ± 5.99^a^	17.95 ± 5.14^a^
abiotic-SeNPs (0.1 ppm)	96.66 ± 5.77^a^	4.1 ± 1.52^a^	27.77 ± 5.71^a^	20.74 ± 4.79^a^
chemical-SeNPs (0.1 ppm)	96.66 ± 5.77^a^	4.14 ± 1.38^a^	27.14 ± 7.61^a^	20.91 ± 5.06^a^
SeO_3_^2−^ (1 ppm)	60 ± 10^b^	2.61 ± 1.33^b^	20.62 ± 5.96^b^	12.97 ± 5.29^b^
abiotic-SeNPs (1 ppm)	96.66 ± 5.77^a^	4.71 ± 1.72^ab^	29.38 ± 5.96^a^	21.89 ± 5.01^a^
chemical-SeNPs (1 ppm)	93.33 ± 5.77^a^	4.79 ± 1.42^ab^	29.57 ± 6.08^a^	22.21 ± 5.06^a^
SeO_3_^2−^ (10 ppm)	13.33 ± 5.77^c^	1.75 ± 0.96^c^	4.38 ± 1.08^c^	2.98 ± 1.16^c^
abiotic-SeNPs (10 ppm)	96.66 ± 5.77^d^	7.36 ± 1.33^d^	37.52 ± 6.61^d^	28.83 ± 5.72^d^
chemical-SeNPs (10 ppm)	93.33 ± 5.77^d^	6.93 ± 1.46^d^	35.89 ± 6.59^d^	27.79 ± 5.84^d^
SeO_3_^2−^ (100 ppm)	0^e^	0^e^	0^e^	0^e^
abiotic-SeNPs (100 ppm)	80 ± 10^a^	4.33 ± 1.05^a^	25.53 ± 5.04^a^	18.49 ± 4.71^a^
chemical-SeNPs (100 ppm)	83.33 ± 5.77^a^	4.53 ± 1.41^a^	27.97 ± 5.08^a^	20.766 ± 4.66 ^a^

Data are means ± standard deviation, means followed for the same letters in the columns do not differ statistically by Kruskal–Wallis test, *p* < 0.05).

The results show that abiotic-SeNPs can be a substitute for chemical-SeNPs. The use of abiotic-SeNPs over chemical-SeNPs is justified because even though their performance is similar, the former has more advantages over nanoparticles from chemical reduction methods. Abiotic reduction is economical, more eco-friendly and safe, unlike chemical reduction techniques requiring downstream processing of hazardous by-products^48^.

Biofortification of plant foods by gradual Se release from SeNPs can be a way to minimise potential losses which can occur if commercial fertilizers are used. This is because SeNPs have been shown to have a substantially positive effect on seed germination^55^. Moreover, there is likely an uptake and translocation of the SeNPs within the *P. sativum* seeds similar to what was observed in a study by Hu et al.^56^ on wheat seedlings. In summary, plants are more resilient to drought, disease and pests because of them^57^.

**Figure 7:**
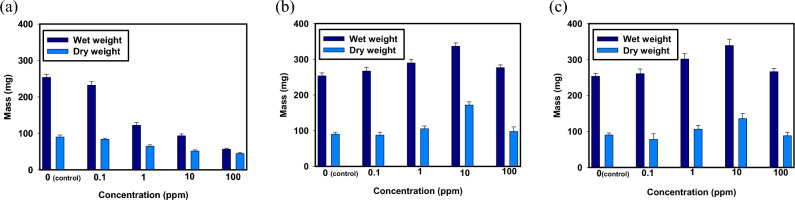
Wet and Dry weight results for varying concentrations of (**a**) Selenite (**b**) Abiotic-SeNPs and (**c**) Chemical-SeNPs.

The wet and dry weight measurements were conducted to assess the effects of different concentrations of selenite, abiotic-SeNPs and chemical-SeNPs on plant growth and biomass accumulation, with a control group serving as the reference. The wet weight of the control group was recorded as 253.6 ± 8.7 mg, while the dry weight was 90.13 ± 5.3 mg. The results are depicted in Fig. 7.

Comparing the selenite-treated seeds, it was observed that as the selenite concentration increased from 0.1 to 100 ppm, both the wet and dry weights showed a consistent decrease. At 0.1 ppm selenite, the wet weight decreased by approximately 8.3% relative to the control, and the dry weight decreased by around 9.4%. The decrease in weight became more substantial with higher concentrations of selenite. At 1 ppm, the wet weight decreased by approximately 51.9%, and the dry weight decreased by about 28.1% relative to the control. Further, at 10 ppm and 100 ppm selenite concentrations, the wet weight decreased by approximately 63.1% and 77.7%, respectively, while the dry weight decreased by around 43% and 49.5% relative to the control. These findings indicate a clear negative impact of selenite on plant growth and biomass accumulation, with higher concentrations leading to more pronounced decreases in weight.

At 0.1 ppm of abiotic-SeNPs, the wet weight increased by approximately 5.3% compared to the control, while the dry weight increased by approximately 2.6%. Moving to 1 ppm abiotic-SeNPs, the wet weight showed an increase of approximately 14.1% relative to the control, and the dry weight increased by approximately 16.8%. Further, at 10 ppm abiotic-SeNPs, the wet weight exhibited an increase of approximately 32.6% compared to the control, while the dry weight increased by around 46%. At the highest concentration tested, 100 ppm abiotic-SeNPs, the wet weight increased by approximately 8.9%, and the dry weight increased by approximately 8.6% relative to the control. These findings suggest a trend of increased wet and dry weights with increasing concentrations of abiotic-SeNPs, with the most substantial increases observed at 10 ppm. The abiotic-SeNPs treatment appears to promote growth and biomass accumulation, although higher concentrations may have diminishing effects on weight gain compared to the optimal 10 ppm concentration.

The wet and dry weight measurements were also performed for the chemical-SeNPs-treated seeds at various concentrations, with the control group serving as the reference. At 0.1 ppm of chemical-SeNPs, the wet weight increased by approximately 2.7% compared to the control, while the dry weight increased by approximately 14.4%. Moving to 1 ppm chemical-SeNPs, the wet weight exhibited an increase of approximately 15.7% relative to the control, and the dry weight increased by around 37.9%. At 10 ppm chemical-SeNPs, the wet weight showed an increase of approximately 33.3% compared to the control, while the dry weight increased by approximately 48.6%. At the highest concentration tested, 100 ppm chemical-SeNPs, the wet weight decreased by approximately 4.3%, and the dry weight decreased by approximately 2.7% relative to the control. These findings indicate a trend of increased wet and dry weights with increasing concentrations of chemical-SeNPs, with the most substantial increases observed at 10 ppm. However, at the highest concentration tested, there was a slight decrease in weight compared to the control. This suggests that while chemical-SeNPs can promote growth and biomass accumulation at lower concentrations, higher concentrations may lead to diminishing effects on weight gain or potential adverse effects on the plant's physiology.

In summary, the wet and dry weight decreased with increasing selenite concentration (0.1–100 ppm), indicating adverse effects on germination, growth, and biomass accumulation^58^. However, a 2020 study on the effect of different forms of selenium on wild peach showed inverse results^59^. This can be an indication that the type of plant and how it utilizes selenium can have a bearing on how it is affected. The highest wet and dry weight values were observed at 10 ppm for both types of SeNPs, suggesting optimal growth. However, concentrations above 10 ppm, including 100 ppm, may result in decreased dry weight due to excessive exposure or potential toxicity^60^. Overall, selenite treatment reduced wet and dry weights at higher concentrations, while 10 ppm of both biogenic SeNPs and chemical SeNPs promoted optimal growth with diminishing effects at higher concentrations.

## Antioxidant activity

**Figure 8:**
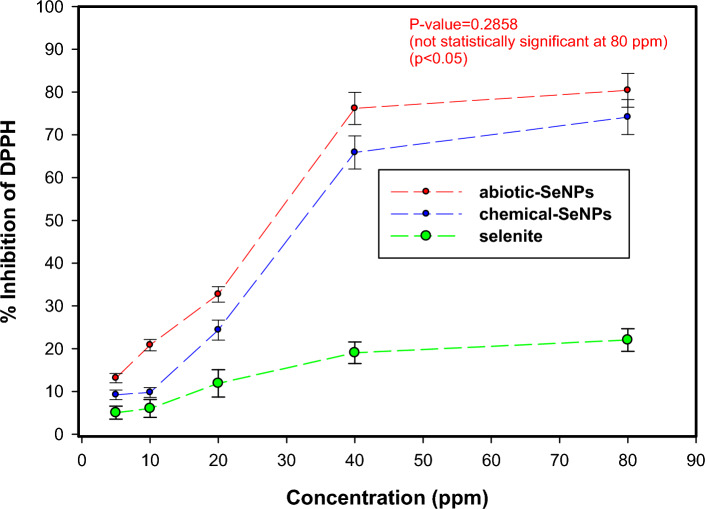
Antioxidant activity of SeO_3_^2−^, abiotic and chemical SeNPs analysed by DPPH assay.

Antioxidants protect plants from stresses such as salinity and drought and they form part of a defence mechanism that prevents the toxic effects of free radicals to seeds^61^. The antioxidant activity is shown in Fig. 8.

The DPPH assay showed that the SeNPs have antioxidant activity (Fig. 8) and the activity of DPPH radical scavenging increased with increasing the concentration of SeNPs. Moreover, the assay went on to indicate that abiotic-SeNPs had a comparable antioxidant activity to chemical-SeNPs with slight differences in performance. When abiotic-SeNPs concentration grew from 5 to 80 ppm, the percentage inhibition increased significantly from 13.13 ± 1.09% reaching 80.41 ± 3.98%. Likewise, when the concentration of the chemical-SeNPs grew with the same magnitude, the percentage inhibition increased significantly from 9.06 ± 1.11% reaching 74.42 ± 4.28%.

The slight differences in the antioxidant activity of the two types of SeNPs may be due to the biomolecules involved in the formation of abiotic-SeNPs^62^ as confirmed by the FTIR and XPS analysis. To add on, besides the presence of the biomolecules, the high ability of SeNPs to deactivate these free radicles might also be due to the dispersibility of nanoparticles through the media owing to the small particle size^33^. Selenite (SeO_3_^2–^) however did not follow the same trend of a large increase in the antioxidant activity in response to increase in its concentration. Between 5 and 80 ppm of sodium selenite, the percentage inhibition changed from 5.04 ± 1.51% reaching 22.03 ± 2.65% which is insignificant as compared to the SeNPs.

It is evident that SeNPs possess better antioxidant capability than other chemical forms of Se but also at the same time reducing the risk of Se toxicity^5^. In addition to the results from this study i.t.o. selenite vs SeNPs toxicity, another study demonstrated antioxidant properties of SeNPs that showed lower toxicity than selenomethionine^63^. This further validates the observations from the germination experiments in which the SeNPs which have high antioxidant activity contributed to seed germination. This could be attributed to the antioxidants’ bio-regulator effects on physiological and biochemical processes in plants. These include; protein synthesis, ion uptake, cell elongation and division, which may the seed quality^61^.

To test the significance between the means at different concentrations of either SeO_3_^2−^, abiotic-SeNPs or chemical-SeNPs test solutions, a two-way ANOVA was used. Table ”5 below summarises the results;

**Table 5:** Two-way ANOVA for determining differences between means for antioxidant activity.

Test solutions	5 ppm	10 ppm	20 ppm	40 ppm	80 ppm
SeO_3_^2−^	5.037 ± 1.5^a^	6.01 ± 2.08^a^	11.90 ± 3.21^a^	19.03 ± 2.52^a^	22.03 ± 2.65^a^
abiotic-SeNPs	13.12 ± 1.09^b^	20.85 ± 1.34^b^	32.71 ± 1.81^b^	76.18 ± 3.77^b^	80.41 ± 3.98^b^
chemical-SeNPs	9.07 ± 1.11^c^	9.62 ± 1.11^c^	24.97 ± 2.64^c^	66.24 ± 4.1^c^	74.41 ± 4.28^b^

Data are means ± standard deviation, means followed for the same letters in the columns do not differ statistically by Two-way ANOVA, *p* < 0.05).

The results indicate that the means for the concentrations are statistically different between 5 and 40 ppm. In each case, the F-statistic was greater than F-critical accompanied by a small *p*-value (1.64 × 10^–13^). At 80 ppm however, F-statistic was less than F-critical and the p-value was larger than the significance level (0.2858). From the results, it can be concluded that the means for the abiotic-SeNPs and chemical-SeNPs are not statistically significant. Therefore, at larger concentrations, the antioxidant activity of the nanoparticles becomes almost similar whereas that for SeO_3_^2−^ does not change significantly.

The similarity in antioxidant activity observed at higher concentrations for abiotic-SeNPs and chemical-SeNPs carries multifaceted implications. Firstly, it implies functional equivalence, suggesting both types possess comparable reactive oxygen species (ROS) scavenging efficacy, thereby mitigating oxidative stress similarly. A study by Sentkowska and Pyrzyńska^64^ found that the antioxidant activity does not always depend only on the SeNPs size but also on their homogeneity. Furthermore, this finding holds environmental significance, as it indicates that abiotic-SeNPs may offer a greener production method, reducing reliance on toxic chemicals and waste by-products associated with chemical synthesis. The former is considered the most environmentally-friendly approach^65^. This shift toward eco-friendlier production can have positive environmental ramifications. Additionally, regulatory considerations come into play, as discerning between abiotic-SeNPs and chemical-SeNPs in safety assessments and regulations may be necessary, especially if their antioxidant activities at higher concentrations continue to align. This could necessitate a re-evaluation of how these nanoparticles are categorised and regulated^66^.

## Conclusion

The study demonstrated how bacteria can be utilised to extract and recover nano-Se by reducing toxic Se oxyanions such as selenite to the less toxic and inert elemental Se nanoparticles. The conducted experiments indicate that Se is more toxic in its anion form as compared to its elemental form because the former hindered seed germination even at minute concentrations (1 ppm). SeNPs were beneficial to seed germination as indicated by the high germination index at 10 ppm. To add on, the SeNPs were found to have higher antioxidant activity which benefits plant germination and can thus be used as an additive in fertilisers. Abiotic-SeNPs had a comparable or at times even better performance to chemical-SeNPs (which are from a costly method). This shows the emergence of bacterial mediated methods slowly replacing already established methods in the race for sustainability. XPS and FTIR results confirmed the presence of elemental Se (Se^0^) and other functional groups which cover the SeNPs. Findings from this study will help further research on sustainable Se recovery and application in the bio-mining arena.

## Data Availability

The datasets generated during and/or analysed during the current study are available from the corresponding author on reasonable request.
